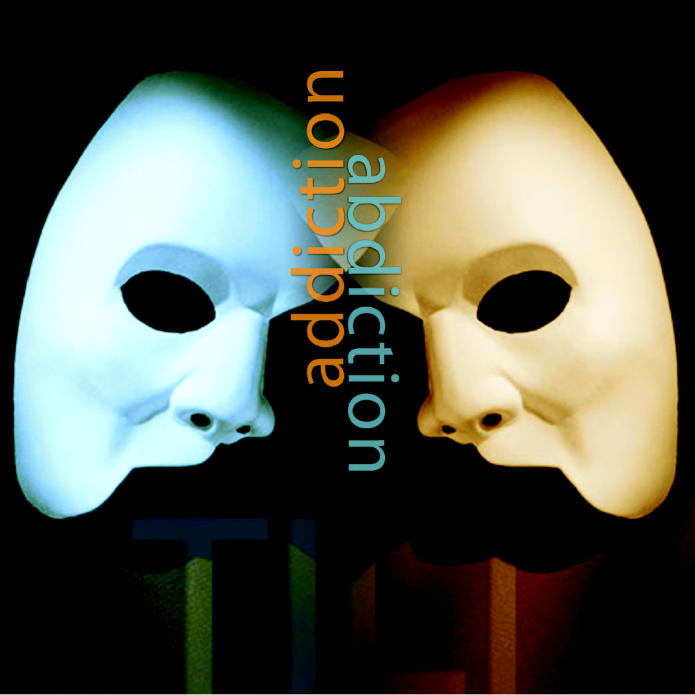# Abdiction/Addiction Connection

**DOI:** 10.1289/ehp.113-a812

**Published:** 2005-12

**Authors:** Ernie Hood

Sometimes in science, as in politics, connections arise that may at first glance appear to be strange bedfellows. That might be the natural first impression of a potential association between chemical intolerance and addiction. But although the conditions are manifested by behaviors that appear to be polar opposites—substance avoidance (or abdiction, as some are beginning to call it) by the chemically intolerant, and compulsive substance use by the addicted—there is evidence to suggest that, biologically, they may actually have much in common.

That was the concept behind “Addiction and Chemical Intolerance: A Shared Etiology?” This conference, held 19–20 September 2005 in Research Triangle Park, North Carolina, was the first scientific meeting to be cosponsored by the NIEHS and the National Institute on Alcohol Abuse and Alcoholism (NIAAA). It was also the first time researchers from the fields of environmental health and addiction convened to explore common ground and potential collaborations.

“The idea of hosting a conference on chemical intolerance and addiction stems from a long history of individual physicians’ reporting observations on patients that looked like addiction to chemicals, foods, caffeine, or alcoholic beverages,” explained conference chair Claudia Miller, a professor and researcher in environmental medicine at The University of Texas Health Science Center at San Antonio. “There is a striking resemblance between the symptoms and responses to substances reported by chemically intolerant patients and individuals addicted to drugs or alcohol.”

Firm numbers on addiction and chemical intolerance are hard to come by, in part because both conditions often go undiagnosed. Approximately 67% of all Americans drink alcohol, yet 90% of the alcohol is consumed by only 30% of the population, said NIAAA director Ting-Kai Li in his keynote address. In the latter half of 2003 (the most recent year for which figures are available), there were 627,923 drug-related emergency room visits in the United States, according to the Drug Abuse Warning Network of the U.S. Substance Abuse and Mental Health Services Administration. As for chemical intolerance, epidemiologic figures compiled and reported at the meeting by William Meggs, a professor of emergency medicine at East Carolina University, suggest the prevalence of the condition (self-reported) to be approximately 12% of the U.S. population, with approximately 4% self-reporting as “seriously affected.”

Miller contends that addiction and chemical intolerance represent divergent physiologic responses to a shared underlying disease mechanism she calls toxicant-induced loss of tolerance (TILT). In TILT, a chemical exposure—either acute or chronic and low-level—initiates sensitization to even small amounts of structurally diverse chemicals found in foods, drugs, alcoholic and caffeinated beverages, pesticides, mold toxins and other elements of indoor air, implanted devices, solvents, cleaning chemicals, and more. Thereafter, when affected individuals are exposed to everyday “triggering” substances such as foods, traffic exhaust, or fragrances, they report multisystem symptoms including headache, nausea, difficulty breathing, muscle spasms, and rashes. The fact that different people exhibit different constellations of symptoms has made it difficult to conduct epidemiologic studies or arrive at a case definition, Miller says. In the past, these difficulties have led some observers to speculate that chemical intolerance is psychogenic in origin.

As she outlined in her presentation to the approximately 120 attendees, Miller postulates that the TILT mechanism can lead to either abdiction or addiction, with both behaviors intended to avoid unpleasant withdrawal symptoms. She further proposes that TILT may underlie a wide variety of chronic diseases that are increasing in prevalence worldwide, such as asthma, autism, chronic fatigue syndrome, fibromyalgia, and depression. (She described these proposals in depth in an article in the January 2001 issue of *Addiction*.)

## Parallel Paths

Whether or not chemical intolerance and addiction are flip sides of the same coin, it is clear that researchers in the two fields have much to learn from each other. Li said, “Some people become alcohol-dependent and then they recover because the environmental risks have been removed; there’s a gene–environment interaction. I think it’s true also for chemical intolerances. It’s an environmentally induced condition, and when you remove the environmental risk, the person may still be genetically high-risk, but without the environmental component they can then recover.”

As things stand today, however, there are no easy answers for the chemically intolerant. Environmental epidemiologist Howard Hu daily perceives the need for more research in his role as a clinician at the Harvard School of Public Health. “Our environmental medicine clinic has several hundred patients who have this disorder, and we have not made any progress in ways to evaluate and manage them that has led to any sustainable improvements in their condition,” he said. “So we really appreciate the need for good research that will shed light on the biology of the disorder and allow us to devise methods to manage and treat it.”

Hu felt that the conference was a good step forward in helping to define a research agenda. “Some of the approaches to chemical addiction and alcoholism [research] have provided a roadmap of where the chemical intolerance research needs to go, in terms of understanding genetic susceptibility and the molecular changes that might be the mechanism of how the intolerance phenotype develops,” he said. One role model for progress described by Miller might be the Japanese government, which has established several environmentally controlled medical units (EMUs) in hospitals for the research, diagnosis, and treatment of chemical intolerance. To date, there is no comparable facility in the United States.

One speaker called attention to “tantalizing morsels” of convergence that have emerged between chemical intolerance and addiction. For example, it appears all but certain that genetic susceptibility plays an important role in both conditions, and one of the most compelling ideas to emerge was the possibility that susceptibility to both conditions may arise from polymorphisms in the same genomic neighborhood—genes including *CYP2D6*, *PON1*, and others that are known to regulate the metabolism of exogenous agents such as drugs and pesticides. *PON1* is involved in the detoxification of organophosphate pesticides; *CYP2D6* functions in the metabolism of structurally diverse substances that affect the central nervous system, including various classes of antidepressants, amphetamines, codeine, and neurotoxicants. The question of whether variant alleles of these genes give rise to the abdiction and addiction phenotypes is a primary target for investigation in the future.

There is a striking resemblance between the symptoms and responses to substances reported by chemically intolerant patients and individuals addicted to drugs or alcohol.

–Claudia Miller

The University of Texas Health Science Center at San Antonio

For now, case–control study results presented by researchers Cornelia Baines and Gail McKeown-Eyssen of the University of Toronto (which were published in the October 2004 *International Journal of Epidemiology*) clearly show an elevated risk for chemical intolerance associated with variations in the enzymatic metabolism genes *CYP2D6*, *PON1*, and *NAT2*. A gene–gene interaction detected between *CYP2D6* and *NAT2* suggested that rapid metabolism alleles in both genes may confer as much as an 18-fold elevated risk for chemical intolerance. These findings point toward a biologic basis for the condition.

Brain imaging studies presented at the conference by Hu, Marc Potenza of the Yale University School of Medicine, and Leonid Bunegin of The University of Texas Health Science Center at San Antonio showed striking similarities between chemically intolerant patients and addicted individuals in terms of the neural regions involved and the types of activation detected. Many signs point to the mesolimbic system, where the activity of neurotransmitters such as dopamine is regulated. Among individuals who are genetically susceptible to either chemical intolerance or addiction, the homeostasis of the brain’s reward system may be upset or perhaps changed permanently by exposures to certain drugs or chemicals. Thus, although the outcomes of addiction and abdiction may be polar opposites, the underlying causes and mechanisms may prove to be very similar.

## You Say Tomato . . .

Differences in nomenclature often pose a challenge and require reconciliation when two fields begin to work together. As one conference presenter waggishly put it, “Scientists would rather use each other’s toothbrushes than use each other’s terminology.”

Perhaps the best example of varying terminology arose as speakers from both fields presented some of the leading hypotheses in each field. In chemical intolerance, researchers refer to “initiation” (the exposure that leads to the development of intolerance) and “triggering” (subsequent exposures resulting in symptoms); in addiction research, scientists refer to neurologic “sensitization” to a substance leading to “amplification” of its effects.

According to Miller, future research may show that neurologic sensitization also explains initiation and triggering. “Perhaps the processes [underlying addiction and chemical intolerance] are one and the same, but we don’t know that quite yet,” she says. “Eventually, once the biology has been worked out, the terminology may reconcile, clarifying the links between the two fields. It was one of the most striking parallels to emerge from the meeting.”

Another impediment discussed during the proceedings is the longstanding struggle to precisely define phenotypes of chemical intolerance for research purposes. Single-minded focus on this difficulty in the past has been the excuse for doing no research, said Miller, who added that facilities like Japan’s EMUs could be used to assess individual responses in the absence of any consensus on case definitions or phenotypes. “Just as there is no single case definition or phenotype that encompasses all forms of drug and alcohol addiction, there is no single case definition that can be applied to all forms of abdiction, because we are dealing with a general mechanism for new classes of diseases that have varied manifestations,” she explained.

Establishing a chemical intolerance phenotype or case definition is further complicated by a phenomenon called “masking.” Underlying chemical or food triggers may be masked by overlapping symptoms resulting from simultaneous or sequential exposures to other foods or chemicals, from addiction to caffeine, alcohol, or tobacco, and from varying degrees of habituation to triggering substances. For example, Miller wrote in her *Addiction* paper, “[i]f an individual is sensitive to many different substances, then the effects of everyday exposures to chemicals, foods, or drugs may overlap, producing a confusing array of symptoms. The individual would feel sick most of the time, but the effect of any single exposure would not be apparent to either the individual or his physicians.” Masking therefore confounds diagnosis and treatment because clinicians tend to address patients’ overt symptoms without discovering the underlying intolerances, much less the initiating exposures that led to illness in the first place.

The lack of phenotypes may also hamper the application of systems biology to the study of chemical intolerance. Systems biology integrates tools from genomics, proteomics, metabolomics, and informatics to detect and validate novel biomarkers of disease. “Without a phenotype, it’s difficult to move to the next level,” said William Slikker, Jr., deputy center director for research at the National Center for Toxicological Research. First, he suggested, we still need to define phenotypes in a way in which they can be systematically examined. “Once that is done,” he said, “then I can see setting hypotheses that can be tested using the systems biology approach.”

At the same time, the availability of a research EMU—the equivalent of a detox unit for alcohol or drug withdrawal—would provide a unique tool for examining individuals’ genetic and protein expression before and after removal of chemical and food triggers and before and after specific challenges, said Miller. “Just as systems biology will enable researchers to understand individual responses to complex environments, the EMU is a tool that [would allow] us to identify the responses of individuals to a wide variety of exposures,” she said.

Miller said the approaches are completely compatible and complementary. “A clear advantage of the EMU is that it can be done now—well before sophisticated genomic and proteomic approaches become widely available—and begin to benefit patients with a wide variety of environmentally induced illnesses.”

## The Road Ahead

NIEHS deputy director Samuel Wilson, who opened the meeting, agreed that the future of the field depends largely on researchers’ ability to carefully identify researchable questions. “It’s going to be up to the scientists writing the proposals or bringing the problems forward to figure out experimental themes or researchable problems that they can make a case for, and then work up and make solid discoveries on,” he said. “There’s no substitute for having quantitative traits to look at—quantitative biochemical markers or biomarkers that can be related with exposure and with these very complex behavioral phenotypes.”

Wilson added that only when the molecular science embedded in the pathophysiology and biology of chemical intolerance and addiction is uncovered will the extent of overlap between the two conditions be established.

Several attendees expressed great interest in pursuing collaborative projects with colleagues from the other field, and many were optimistic that the conference would ultimately result in cross-institute initiatives between the NIEHS and NIAAA. For environmental health researchers, addiction has long been a blind spot; in addiction research, the same is true for environmental exposures. With greater interactions between the two fields, both may achieve a clearer view of these conditions and the road to health.

## Figures and Tables

**Figure:**